# Association of maternal late-gestation lipid mobilization and their offspring's disease risk during the pre-weaned period and performance through first lactation: A cohort study in a dairy herd

**DOI:** 10.3389/fvets.2023.1102421

**Published:** 2023-02-23

**Authors:** Ana Velasquez-Munoz, Emily J. Schuurmans, Jill L. Brester, Kathryn Starken, Angel Abuelo

**Affiliations:** Department of Large Animal Clinical Sciences, College of Veterinary Medicine, Michigan State University, East Lansing, MI, United States

**Keywords:** calf health, fetal programming, metabolic stress, transition period, lipid mobilization

## Abstract

**Introduction:**

Excessive maternal lipid mobilization in late gestation may impact the immune function of the newborn. However, the long-term effects remain unknown. The objective was to explore associations between excessive maternal lipid mobilization in the last 2 weeks of gestation with offspring health and performance.

**Methods:**

A retrospective study was performed including 1,511 calves (heifer = 692, bull = 819) born between 2015 and 2020 in one MI farm. Plasma non-esterified fatty acids (NEFA) was measured from cows 7 to 14 d before calving. Calves were categorized in 2 groups based NEFA concentration: physiological lipid mobilization (PLM = 1,373; NEFA <0.3 mM) and excessive lipid mobilization (ELM = 138; NEFA ≥0.3 mM). Calf records were obtained from the herd's management software. Outcomes of interest were the hazard of pre-weaned digestive and respiratory disease, pre-weaned ADG, age at first breeding and calving, first lactation 305 d mature equivalent milk yield (305ME), and survival until first calving. Statistical models included dam NEFA category adjusted by year and season of birth, parity of the dam, and sex of the calf. Cox proportional analysis was used to determine the hazard of a pre-weaned health event, first breeding, and first calving. Linear regression was used to evaluate ADG and 305ME. The survival until first calving was analyzed with logistic regression.

**Results and discussion:**

No difference was detected in the hazard of diarrhea (HR_PLM vs. ELM_ = 1.06; 95% CI = 0.82–1.38) and respiratory disease (HR_PLM vs. ELM_ = 1.29; 95% CI = 0.79–2.10) by NEFA category in the pre-weaned period. Also, no difference was detected for the LSM (±SE) of pre-weaned ADG (PLM = 0.77±1.55, ELM = 0.72±2.76 kg/d). In heifers, the hazard for first breeding favored the PLM group (HR_PLM vs. ELM_ = 1.59; 95% CI = 1.18–2.12), with a reduced median age at first breeding (PLM = 400 d, 95% CI = 397–402; ELM = 412 d, 95% CI = 404–421). However, NEFA category was not associated with the hazard of first calving (HR_PLM vs. ELM_ = 0.94; 95% CI = 0.69–1.27), first lactation 305ME (PLM = 16,665±165 kg; ELM = 16,256±532), the odds of presenting at least 1 health event in the first lactation (OR_PLM vs. ELM_ = 0.78; 95% CI = 0.41–1.49), or the odds of leaving the herd before first calving (OR_PLM vs. ELM_ = 1.21; 95% CI = 0.56–2.02). Overall, dam ELM affected the hazard of first breeding but no other indicators of health or long-term performance. However, associations between maternal lipid mobilization and calf outcomes cannot be excluded, as the NEFA cut-off used has not been established as a predictor of offspring health and performance.

## 1. Introduction

Dairy cows experience a period of energy deficit (ED) in the transition from late gestation to early lactation due to the increased demands for fetal growth, the onset of milk production, and decreased DMI (1). Lipid mobilization is a physiological adaptation to offset ED. Lipolysis results in the release of non-esterified fatty acids (NEFA) from the adipose tissue to the bloodstream as a form of readily available energy substrate (2). In a balanced metabolic response, NEFA initiates negative feedback to modulate the extent of lipid mobilization and maintain a stable glucose concentration to satisfy the energy needs of the transition cow (3). However, excessive release of lipids and accumulation of NEFA might compromise the inflammatory responses and ultimately modify the cow's immune function (2). The quantification of plasma NEFA is used as a diagnostic method to monitor the metabolic status of the transition cow. A NEFA concentration greater or equal than 0.3 mM in the last 14 to 3 d of gestation has been classified as excessive lipid mobilization, and it has been linked to a greater risk for post-partum disease events, early lactation culling, and reproductive performance (4–6).

Excessive lipid mobilization (ELM) can start pre-calving and might, therefore, affect the fetus. However, despite the evidence linking ELM with impaired immune responses and increased disease risk in the dam, there is limited information about how the *in utero* exposure to the elevated NEFA concentrations released during ELM might affect the fetus and offspring in the short and long term. Furthermore, excessive lipid mobilization is a hallmark of metabolic stress (3). Evidence suggests a potential association between exposure to maternal metabolic stress and excessive NEFA in the last 28 days of gestation with an increase in serum haptoglobin, TNFα, oxidant status index of the calf, less robust LPS-induced inflammatory response, and reduced birth weight that last for the first 4 weeks of life (7). Although, those authors did not evaluate if the changes in the immune status of the neonatal calf were associated with an impairment of health and performance during the pre-weaned period or later in life. However, maternal stressors in the last third of gestation are well known for exerting carryover effects on the offspring's neonatal life and adulthood. For example, exposure to *in-utero* heat stress during the dry period has been associated with decreased growth rates, impaired immune responses, early culling, and reduced milk yield in heifer calves (8–10). Likewise, calves that experienced prenatal nutritional restriction had a reduced birth weight, impaired immunity, and antioxidation capability at birth (11).

To our knowledge, there is no evidence demonstrating the impact of exposure to maternal lipid mobilization in the last weeks of gestation on the risk of diseases or on the long-term performance of the calf. Thus, the objective of this retrospective cohort study was to explore associations between excessive lipid mobilization prepartum (plasma NEFA ≥ 0.3 mM) and the health, reproductive, and productive performance of the offspring until the first lactation. We hypothesized that excessive maternal lipid mobilization in the last 2 weeks of gestation is associated with greater risk for neonatal disease, lower ADG, and impaired productive and reproductive performance.

## 2. Materials and methods

### 2.1. Farm management

A retrospective cohort study was conducted using herd records of a large commercial dairy farm associated with the Michigan State University Training Center for Dairy Professionals (Elsie, MI). The farm had approximately 3,500 lactating Holstein cows with a rolling herd average milk production of 12,250 kg/cow. In this facility, heifer and bull calves were managed identically from birth to weaning.

The management of the newborn calf included the administration of an oral vaccine (Calf-Guard, Zoetis Services) against diarrhea pathogens, an intranasal vaccine (Inforce 3, Zoetis Services) against respiratory pathogens, and a vitamin E and selenium complex subcutaneously (MU-SE, Merck Animal Health) after birth. After 30 min, calves were weighed with a scale and fed 3 L of >22% Brix fresh or frozen colostrum *via* an orogastric tube. As part of the weekly farm practices, a random sample of calves is selected for the measurement of serum total protein for evaluation of transfer of passive immunity. During the study period, the weekly prevalence of poor transfer of passive immunity category (total protein ≤ 5.1 g/dL) ranged between 0 and 6.8%. Thus, the effectiveness of the farm's colostrum feeding protocol was adequate as stablished in the new recommendations for passive immunity in calves (12).

After birth, calves were housed in individual stalls inside a shed, and they were offered 3 L of milk replacer (28% CP, 20% CF; Cow's Match, Land O'Lakes Inc.) mixed at 15% solids, three times per day in a bucket until weaning. Water and starter concentrate (20% protein, 2.5% fat, 7.5% fiber) were offered *ad libitum*. The starter was introduced at 1 week of age. Calves were weaned using a step-down approach that started gradually decreasing the amount of milk replacer offered at 7 weeks of age for weaning at 10 weeks of age (average: 80, range: 77–85 days). Weaning weight was collected with a scale before calves were moved to the transition barns.

Daily pre-weaned disease diagnosis and treatment were performed by farm personnel following the protocols designed by the herd veterinarians. For this, dedicated farm personnel inspected the calves daily. Calves with clinical signs and those with a reduced milk consumption were examined in detail using the health scoring system developed by McGuirk and Peek (13) to guide treatment decisions. This scoring system classifies the following clinical parameters using a 0–3 scale: rectal temperature, cough, nasal discharge, eyes, ears, and feces. A total respiratory score is also calculated by summing the scores of rectal temperature, cough, nasal discharge, and the highest score of eyes and ear. Diarrhea was defined as having a fecal score ≥2 (loose but staying on top of bedding). Respiratory disease was defined as having a total respiratory score ≥5 or that have two or more respiratory clinical parameters (rectal temperature, cough, nasal discharge, eyes or ears) with score 2 or 3. All treatments were recorded on the herd management software. After weaning, heifer calves were housed in free-stalls pens grouped by age and reproductive status. From 11 month of life, heifers were weighted fortnightly. Heifers older than 12 months and with bodyweight over 365 kg were inseminated by trained personnel based on observed standing estrous. The personnel performed assessments for heat detection in the heifer pens twice per day. The farm protocols allowed up to 8 AI per heifer. Sexed semen was used for the first 2 inseminations and beef-breed semen for subsequent inseminations. Pregnancy was confirmed 30 to 40 days after insemination *via* rectal ultrasonography.

### 2.2. Data collection and sample handling

The farm enrolled in this study conducted pre-calving plasma NEFA analyses every 2 weeks for monitoring transition cow health. For this, ten primiparous and ten multiparous cows were randomly selected (www.graphpad.com/quickcalcs/randomSelect1/) for blood collection among all cows expected to calve in the following 7 to 14 d. Blood samples were collected at the time of daily delivery of fresh total mixed ration. Samples were obtained *via* puncture of coccygeal vessels using EDTA vacuum tubes (Monoject EDTA K3; Covidien, Minneapolis, MN). Immediately after collection, samples were centrifuged at 2,000 x *g* for 10 min. Plasma was removed and placed into 2-mL microcentrifuge tubes (Fisher Scientific, Waltham, MA). All samples were refrigerated, and plasma NEFA concentration was determined within 24 h by Central Star Cooperative (Grand Ledge, MI) using a 96-well plate protocol validated for cattle (14). The test has a sensitivity (95%CI) of 88.9% (67.2–96.9%) and a specificity (95% CI) of 100.0% (97.1–100.0%) for the identification of excessive lipid mobilization pre-calving (14). A convenience sample size that included the results from all the cows sampled between September 2015 and March 2020 whose actual calving date was between 4 and 14 days [mean (SD) = 7.4 (2.1) days] after sampling (*n* = 1,532) were included in the study. The details regarding calving date, calf identification, calf health, and calf performance were extracted from the farm record system (DairyComp 305, Valley Agricultural Software, Tulare CA).

### 2.3. Data management

**Figure 1:**
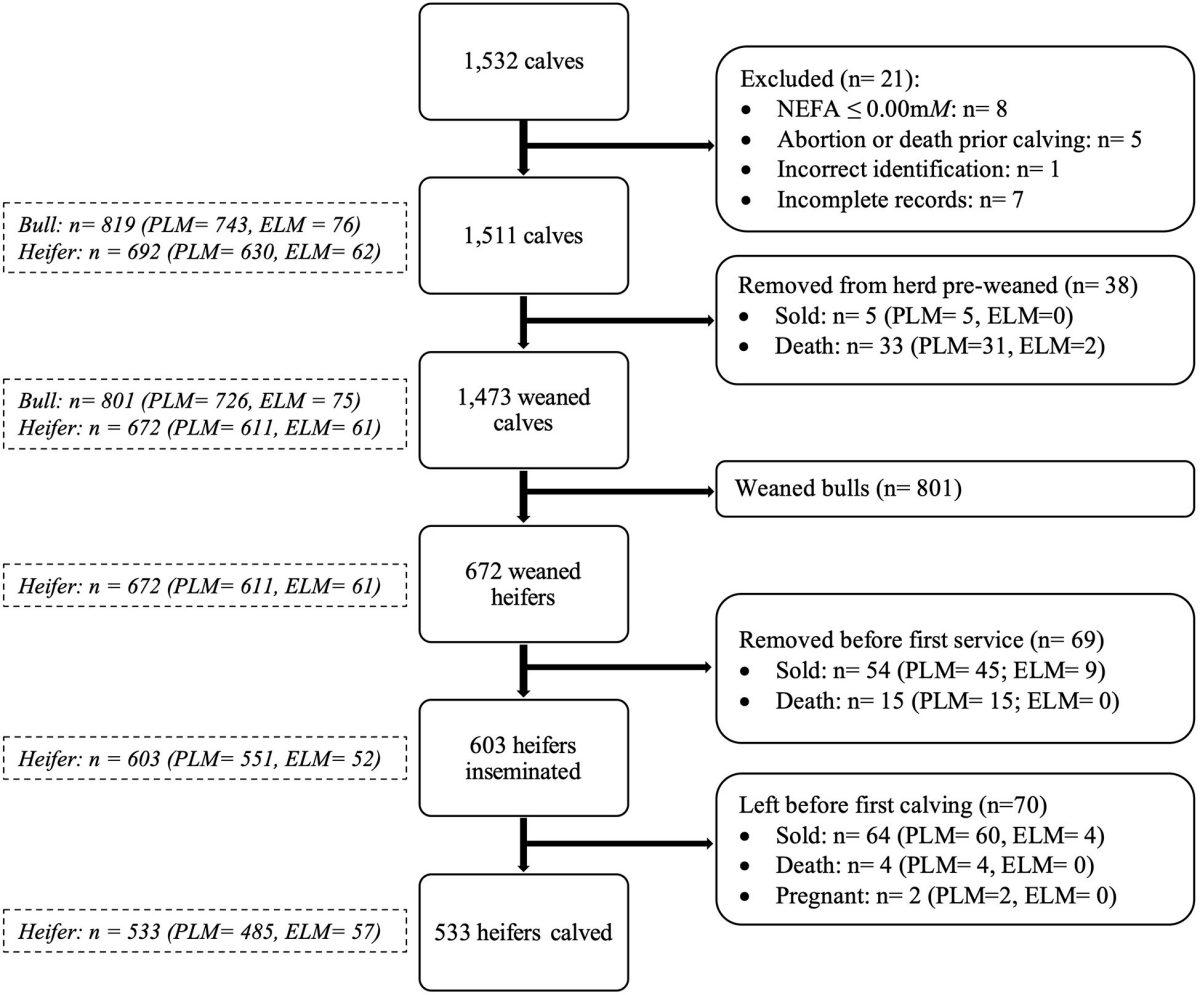
Flow chart describing the number of animals considered in the study (PLM, physiological lipid mobilization; ELM, excessive lipid mobilization).

Plasma NEFA results and calf records were exported into a spreadsheet (Excel, Microsoft Corporation, Redmond WA). Subsequently, records were screened for completeness and accuracy. Eight cows were removed from data analysis because their records showed NEFA concentrations ≤ 0.00 mM, and it was impossible to re-assay these samples. Data from five cows were removed due to abortion or death between sampling and calving. One cow was removed due to incorrect identification in the NEFA database. Moreover, seven calves had incomplete records in the farm's software, and they were removed. Consequently, a total of 1,511 animals were included for analysis (Figure 1).

**Figure 2:**
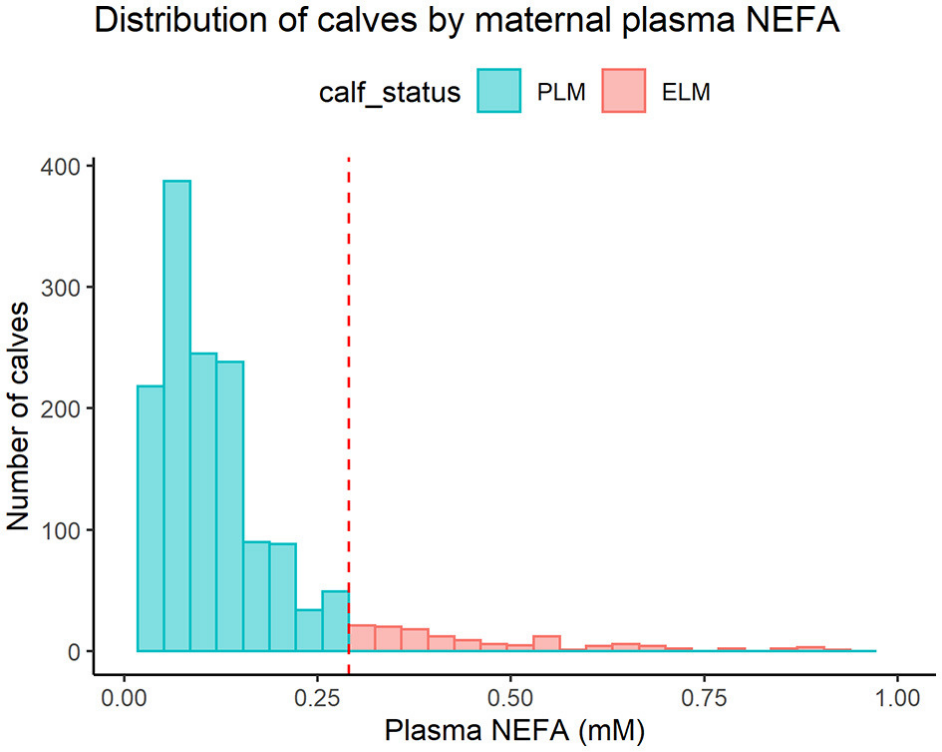
Histogram for the distribution of calves by maternal plasma nonesterified fatty acids (NEFA) category. PLM (physiological lipid mobilization = 1,373; heifers = 630; bulls = 743; plasma NEFA <0.3 mM; blue), ELM (excessive lipid mobilization = 138; heifers = 62; bulls = 76; plasma NEFA ≥0.3 mM; red). The red dashed line represents the NEFA cut-off of 0.3 mM used to define ELM.

Calves were classified based on the status of their dam's plasma NEFA concentrations using the 0.3 mM cut-off for excessive lipid mobilization prepartum (5). Calves born to dams with NEFA concentrations greater or equal to 0.3 mM were included in the Excessive Lipid Mobilization (ELM) group (*n* = 138). Calves born to dams with NEFA concentrations lesser than 0.3 mM were classified in the Physiological Lipid Mobilization (PLM) group (*n* = 1,373). The 25th and 75th percentiles for the ELM dams were 0.35 and 0.58, and for the PLM dams were 0.06 and 0.14, respectively. Furthermore, the median (IQR) of NEFA concentrations of ELM and PLM dams were 0.42 (0.23) and 0.09 (0.08) mM, respectively. Figure 2 shows the distribution of calves by maternal NEFA category.

The ADG for the pre-weaned period was calculated based on the recorded birth and weaning weights. In addition, the date and diagnosis and treatment of a health event was collected from the farm records; thus, the definition of disease was linked to farm treatments and their assessment of health status. Moreover, a new pre-weaned disease treatment was considered when two health records were at least seven days apart. For the analysis, the pre-weaned period was considered as the first 80 days of life. Finally, the season and year of birth were added to the database for each calf. Seasons were described as follows: Summer (June, July, and August), Fall (September, October, and November), Winter (December, January, and February), and Spring (March, April, and May). The records of both male (ELM = 76; PLM = 743) and female calves (ELM = 62; PLM = 630) were included for the analysis of pre-weaned variables. In contrast, only records of heifer calves were used for health, reproductive and productive outcomes until the first lactation (age at first service and calving, survival until the first lactation, number of disease events during the first lactation, and 305ME in the first lactation).

### 2.4. Statistical analyses

All statistical analyses were performed in R (15) with the packages dplyr (16), lubridate (17), survminer (18), survival (19), ggplot2 (20), pscl (21), and emmeans (22). A convenience sample size of 1,511 calves with complete records was included for the analysis (Figure 1). Data were analyzed considering calf as the experimental unit. Additionally, a model was built using the maternal plasma NEFA category as the main predictor adjusted by sex of the calf, the season of birth, year of birth, and dam parity (primiparous/multiparous). Sex of the calf was excluded when only heifer data was analyzed.

Proportional hazard ratios were computed to calculate the hazards of a calf being treated for digestive or respiratory disease during the pre-weaned period, breeding, and first calving. The model assumptions were assessed with the Schoenfeld residual test; variables violating the assumptions were stratified in the model (23). The adjusting variable stratified for the hazard of diarrhea treatment, first breeding and first calving was year of birth. The variables stratified for the hazard of respiratory treatments were calf sex and parity of the dam. In the model, the main predictor was adjusted by the stratified variables, although an estimate was not provided for them. Our interest was to determine the effect of plasma NEFA category in the outcome variables, and not the effect of the adjusting variables. The model assumptions were re-assessed, and the assumptions were met. The Likelihood Ratio Test was used to determine significance. Kaplan Meier analysis was performed to estimate median survival time and obtain a survival curve for each pre-weaned health treatment and reproductive event by maternal plasma NEFA category. Also, a visual representation of the number of treatments for disease events was performed, zero-inflated Poisson regression was used to identify differences in the number of new treatments during the pre-weaned period by NEFA category.

A linear regression model was built to calculate differences in the LSM for birth weight, weaning weight, ADG during the pre-weaned period, number of AI per pregnancy, and 305ME by the NEFA category of the dam adjusted for the variables previously described. Normality and homoscedasticity were assessed with residual plots along with Shapiro-Wilk and Levene's Test. Finally, multiple logistic regression was used to analyze the odds of presenting at least one treatment for disease during the first lactation and the odds of leaving the herd from birth until the first calving by NEFA category. Chi-square was calculated to compare cause of herd removal by NEFA category. Missing data was further analyzed to find patterns not associated to randomness in the records, when random missing values existed, they were excluded from the analysis. A significant level was established at *P* < 0.05.

## 3. Results

### 3.1. Descriptive statistics

The records of 1,511 calves were included in this study. In total, 9.1% (*n* = 138) of the calves were classified as ELM and 90.9% (*n* = 1,373) as PLM by plasma NEFA category of the dam. The mean (SD) dry period length for the ELM and PLM groups were 52.8 (6.6) and 52.9 (5.9) d, respectively. In addition, the dataset included 54.2% (*n* = 819; ELM = 76; PLM = 743) of bull calves and 45.8% (*n* = 692; ELM = 62; PLM = 630) of heifer calves. In total, 44.7% (*n* = 675) and 55.3% (*n* = 836) of calves were born from primiparous and multiparous cows, respectively. The distribution of calves by season of birth ranged between 23.3 and 28.1% (Table 1). A detailed description of calves by NEFA category for calf sex, dam parity, season of birth, and year of birth can be found in Table 1.

**Table 1:** Descriptive statistics for predictor variables organized by maternal plasma NEFA category in the late gestation.

**Variable**	**Maternal Plasma NEFA Category**		**Total, *n* (%)**
	**PLM**, ***n*** **(%)**	**ELM**, ***n*** **(%)**	
**Parity of the dam**			
Primiparous	640 (46.6)	35 (25.4)	675
Multiparous	733 (53.4)	103 (74.6)	836
Total	1,373 (100)	138 (100)	1,511 (100)
**Sex of the calf**			
Bull	743 (54.1)	76 (55.1)	819
Heifer	630 (45.9)	62 (44.9)	692
Total	1,373 (100)	138 (100)	1,511 (100)
**Year of birth**			
2015	103 (7.5)	16 (11.6)	119
2016	394 (28.7)	39 (28.3)	433
2017	431 (31.4)	50 (36.2)	481
2018	217 (15.8)	21 (15.2)	238
2019	176 (12.8)	9 (6.5)	185
2020	52 (3.8)	3 (2.2)	55
Total	1,373 (100)	138 (100)	1,511 (100)
**Season of birth**			
Fall	391 (28.4)	33 (23.9)	424
Winter	322 (23.5)	30 (21.8)	352
Summer	322 (23.5)	38 (27.5)	360
Spring	338 (24.6)	37 (26.8)	375
Total	1,373 (100)	138 (100)	1,511 (100)

PLM (physiological lipid mobilization, plasma NEFA < 0.3 mM), ELM (excessive lipid mobilization, plasma NEFA ≥0.3 mM).

### 3.2. Pre-weaned health and performance

**Figure 3:**
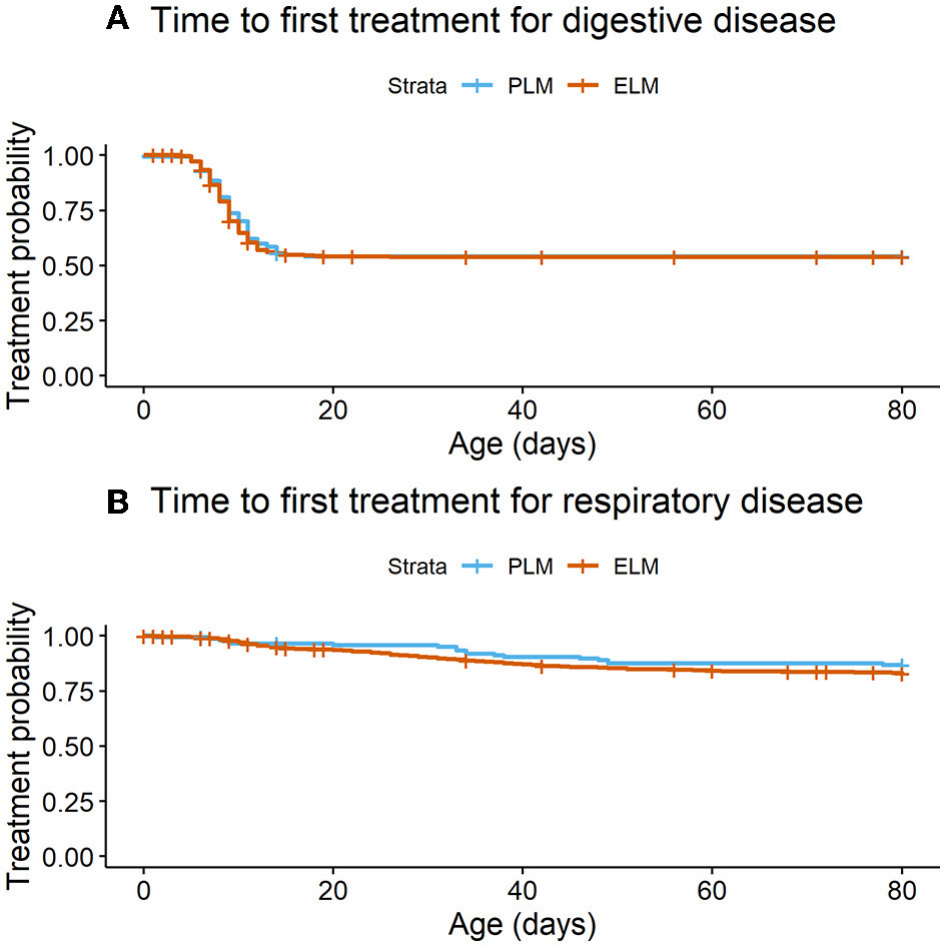
Time to occurrence of diseases in the pre-weaned life (birth to 80d of life) by maternal plasma NEFA category in the last 2 weeks of gestation. PLM (physiological lipid mobilization = 1,373; plasma NEFA <0.3 mM; blue line), ELM (excessive lipid mobilization = 138, plasma NEFA ≥0.3 mM; red line). **(A)** Time to first treatment for digestive disease (*P* = 0.83). **(B)** Time to first treatment for respiratory disease (*P* = 0.23).

The total number of health events by calf in the first 80 days of life ranged from 0 to 4. Where, 46.2% (*n* = 698), 44.0% (*n* = 665), 8.5% (*n* = 129), 1.0% (*n* = 15), and 0.3% (*n* = 4) of the calves presented 0, 1, 2, 3, 4 events, respectively. Enrolled calves did not differ in the treatment rate ratio in the first 80 days of life by NEFA category (Rate ratio_PLMvs.ELM_ = 1.09; 95% CI = 0.87–1.37; *P* = 0.99). Likewise, the mean number of treatments did not differ by maternal NEFA category (Table 3). Overall, 45.6% (*n* = 689) of the calves were treated for diarrhea before weaning (PLM = 45.6%, *n* = 626; ELM = 45.7%, *n* = 63). No association was observed in the hazard of a calf for exhibiting diarrhea in the pre-weaned period by the maternal NEFA category (Table 2, Figure 3A).

**Table 2:** Comparison of health, reproductive, and survival outcomes organized by statistical analysis using an adjusted model^a^.

**Variable**	**HR/OR (PLM vs ELM)**	**95% CI**	***P*-value**
Digestive disease^b^	1.06	0.82–1.38	0.65
Respiratory disease^b^	1.29	0.79–2.10	0.27
First breeding^b^	1.58	1.18–2.12	0.001
First calving^b^	0.94	0.69–1.27	0.69
Disease first lactation^c^	0.78	0.41–1.49	0.44
Left the herd before first calving^c^	1.21	0.56–2.02	0.90

PLM (physiological lipid mobilization, plasma NEFA < 0.3 mM), ELM (excessive lipid mobilization, plasma NEFA ≥0.3 mM). ^a^NEFA adjusted by season of birth, year of birth, dam parity, and sex of the calf in pre-weaned variables. ^b^HR, hazard ratio. ^c^OR, odds ratio.

Furthermore, 16.6% (*n* = 251) of the enrolled calves were treated for respiratory disease before weaning (PLM = 17.0%, *n* = 233; ELM = 13.0%, *n* = 18). The hazard of a calf for receiving treatment for pre-weaned respiratory disease did not differ by maternal NEFA category (Table 2, Figure 3B). In relation to performance, birth weight was collected in 63.6% (*n* = 961) of the enrolled calves. Calves in the PLM category were 1.2 kg heavier at birth than ELM calves (Table 3). Moreover, weaning weight was recorded in 13.2% (*n* = 195) of the calves that survived the pre-weaned period (*n* = 1,473). Weaning weight and pre-weaned ADG did not differ by maternal NEFA category (Table 3).

**Table 3:** Least square means for pre-weaned performance, health, reproductive, and productive parameters by maternal NEFA category in the late gestation.

**Variable**	**Maternal plasma NEFA category**		***P*-value**
	**PLM**	**ELM**	
	**LSM** ±**SE**	**LSM** ±**SE**	
Birth weight (kg)^a^	42.4 ± 0.17	41.2 ± 0.53	0.04
Weaning weight (kg)^b^	92.3 ± 2.51	95.8 ± 1.49	0.14
ADG (kg/d)^c^	0.72 ± 0.03	0.77 ± 0.02	0.10
Number health events (0–80d)	0.66 ± 0.02	0.62 ± 0.06	0.37
Number of AI	2.28 ± 0.06	1.80 ± 0.20	0.31
305ME (kg)^d^	16,665 ± 165	16,256 ± 532	0.11

^a^*n* = 691 (ELM = 89, PLM = 872).

^b^*n* = 195 (ELM = 22, PLM = 173).

^c^*n* = 195 (ELM = 22, PLM = 173).

^d^305ME = 305-d mature equivalent milk production, *n* = 501 (ELM = 44, PLM = 457).

PLM (physiological lipid mobilization, plasma NEFA < 0.3 mM), ELM (excessive lipid mobilization, plasma NEFA ≥0.3 mM).

### 3.3. Heifer reproductive performance

**Figure 4:**
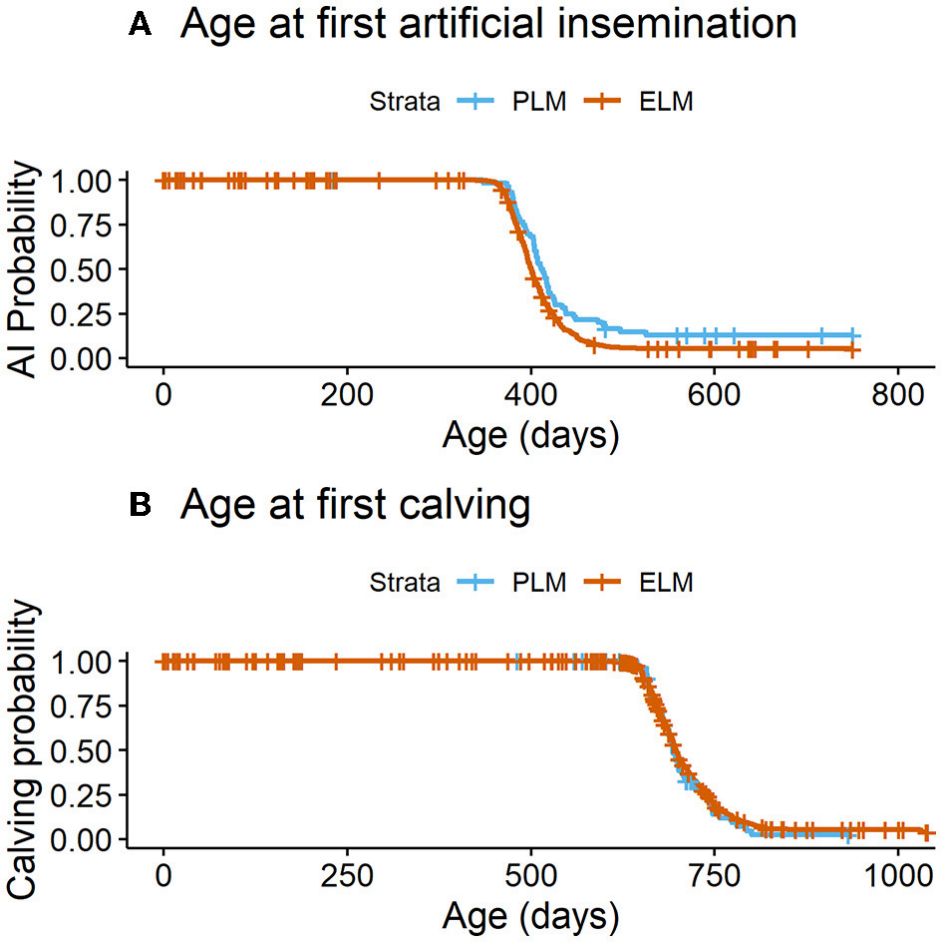
**(A)** Time to the occurrence of the first AI by maternal plasma NEFA category in the last 2 weeks of gestation. PLM (physiological lipid mobilization = 529; plasma NEFA <0.3 mM; blue line), ELM (excessive lipid mobilization = 50; plasma NEFA ≥0.3 mM; red line), *P* = 0.007. **(B)** Time to first calving by maternal plasma NEFA category in the last 2 weeks of gestation. PLM (physiological lipid mobilization = 457; plasma NEFA <0.3 mM; blue line), ELM (excessive lipid mobilization = 45; plasma NEFA ≥0.3 mM; red line), *P* = 0.4.

In total, 87.1% (*n* = 603) of all enrolled heifers had at least one AI (PLM= 87.5%, *n* = 551; ELM = 84.0%, *n* = 52). The median age (95% CI) to first breeding differed by NEFA category (PLM = 400 d (397–402) vs. ELM = 412 d (404-421); *P* = 0.007; Figure 4A). In addition, heifers in the PLM category had 1.6 times greater hazard of having at least one AI (Table 2). Also, the number of inseminations per heifer ranged from 1 to 8, with a median of 2 AI. The mean number of AI to pregnancy did not differ for heifers in the PLM and ELM category (Table 3). Moreover, 81.3% (*n* = 563) of all heifers had a pregnancy confirmation (PLM = 81.3%, *n* = 512; ELM = 82.2%, *n* = 51), and 77.0% (*n* = 533) of heifers had a first calving (PML = 76.9%, *n* = 485; ELM = 77.4%, *n* = 48). No differences were observed in the median age (95% CI) at first calving (PLM = 697 d (694–702); ELM = 693 d (684–710); *P* = 0.4; Figure 4B) or in the hazard of first calving between maternal NEFA categories (Table 2).

### 3.4. First lactation health and milk yield

Among the 533 heifers that reached the first lactation, 194 (PLM = 172; ELM = 22) treatments for disease events were recorded in 177 cows (PLM = 158, ELM = 19), the range of treatments per cow was 1 to 2. The frequency and cause of diseases were 70.1% (*n* = 136) lameness, 12.4% (*n* = 24) metritis, 11.3% (*n* = 22) mastitis, 3.1% (*n* = 6) displaced abomasum, 2.6% (*n* = 5) ketosis, and 0.5% (*n* = 1) milk fever (Table 4). Due to the low frequency of treatments, a comparison by each disease and the NEFA category was not possible. Thus, all treatments, regardless of the cause, were combined in a binary outcome. The odds of a cow to have at least one treatment for disease in the first lactation was not associated with the maternal NEFA category (Table 2). Finally, from the 533 heifers that had a first calving, 93.9% (*n* = 501; PLM= 94.2 %, *n* = 457; ELM= 91.6 %, *n* = 44) had record for 305ME. Maternal NEFA category was not associated with the mean 305ME in the first lactation (Table 3).

**Table 4:** Frequency of diseases in the first lactation by maternal NEFA category in the late gestation.

**Health event**	**Maternal plasma NEFA category**		**Total, *n* (%)**	***P*-value^a^**
	**PLM**, ***n*** **(%)**	**ELM**, ***n*** **(%)**		
Lameness	120 (61.9)	16 (8.2)	136 (70.1)	–
Metritis	21 (10.8)	3 (1.6)	24 (12.4)	–
Mastitis	21 (10.8)	1 (0.5)	22 (11.3)	–
Displaced Abomasum	6 (3.1)	0 (0)	6 (3.1)	–
Ketosis	3 (1.6)	2 (1.0)	5 (2.6)	–
Milk Fever	1 (0.5)	0 (0)	1 (0.5)	–
Total	169 (88.5)	22 (11.5)	194 (100)	0.16

^a^*P*-value for the logistic regression analysis comparing the odds of disease by maternal lipid mobilization group.

PLM (physiological lipid mobilization, plasma NEFA < 0.3 mM, *n* = 457), ELM (excessive lipid mobilization, plasma NEFA ≥0.3 mM, *n* = 45).

### 3.5. Removal from the herd

Overall, 2.5% (*n* = 38; PLM = 36; ELM = 2) of bull and heifer calves left the herd in the pre-weaned period. Deaths were the main reason for leaving the facility in the PLM and ELM group (Table 5). When including only heifer data, 77.3% (*n* = 535; PLM = 487; ELM= 48) of heifer calves reached first calving and 22.7% (*n* = 157; PLM = 143; ELM = 14) left the facility. A total of 121 (PLM = 108; ELM = 13) heifers were sold, and 36 (PLM = 35, ELM = 1) died from birth until the first calving (Table 5). Furthermore, the odds of leaving the herd from birth until the first calving did not differ by maternal NEFA category (Table 2).

**Table 5:** Cause and proportion of calves and heifers that left the herd in the pre-weaned period or before the first lactation by maternal NEFA category in the late gestation.

**Left the herd**	**Maternal plasma NEFA category**		**Total, *n* (%)**	***P*-value**
	**PLM**, ***n*** **(%)**	**ELM**, ***n*** **(%)**		
Deaths 0–80 days^a^	31 (2.1)	2 (0.1)	33 (2.2)	
Sells 0–80 days^a^	5 (0.3)	0 (0)	5 (0.3)	
Total 0–80 days^a^	36 (2.4)	2 (0.1)	38 (2.5)	0.32
Sells 0 days until first calving^b^	108 (15.6)	13 (1.9)	121 (17.5)	
Deaths 0 days until first calving^b^	35 (5.1)	1 (0.1)	36 (5.2)	
Total 0 days until first calving^b^	143 (20.7)	14 (2.0)	157 (22.7)	0.29

^a^Includes heifer and bull calves, *n* = 1,511 (PLM = 1,373, ELM = 138).

^b^Includes only heifer calves, *n* = 692 (PLM = 630, ELM = 62).

PLM (physiological lipid mobilization, plasma NEFA < 0.3 mM), ELM (excessive lipid mobilization, plasma NEFA ≥0.3 mM).

## 4. Discussion

The objective of this retrospective study was to explore potential associations between maternal ELM in the last 2 weeks of gestation and indicators of health and performance in the offspring from birth until the first lactation. Several studies have reported that stressors in late gestation have consequences on the health and performance of the offspring (8, 24, 25). Excessive lipid mobilization is a hallmark of metabolic stress (1), and is usually determined when plasma NEFA concentrations are >0.3 mM pre-partum. Our previous research showed that increased dam NEFA concentrations in late gestation are associated with lower immunological responses in their offspring (7). It is unknown if the current cut-off for excessive plasma NEFA concentration in the transition cow may be associated with disease susceptibility and reduced productivity in the calf. However, in our previous study, cows in the high prepartum NEFA group had a mean (SD) NEFA concentration of 0.37 (0.10) mM, and this level of lipid mobilization was associated with changes in immune responses and inflammatory status of calves (7). Thus, we sought to evaluate, for the first time, a potential association between the NEFA cut-off used to define ELM in transition cows (6) and the offspring's long-term health and performance. For that reason, in the discussion will be included studies focused on diverse stressors in the last trimester of gestation. We acknowledge that the mechanisms behind prenatal stressors differ, and a direct comparison is therefore not warranted or valid. However, it is effective to remark that carryover effects might affect a wide variety of outcomes in the offspring. Finally, it is important to mention that due to the nature of our data, we lost 54% of our animals when bull calves were weaned and excluded from the analysis related to reproduction and production.

### 4.1. Pre-weaned health and performance

In our study, excessive maternal lipid mobilization in the last 2 weeks of gestation was not associated with the hazards of treatments for digestive or respiratory diseases during the pre-weaned period. Urie *et al*. (26) reported a national average for morbidity of pre-weaned digestive and respiratory disease of 18.9 and 11.2%, respectively. The treatments for these two disease events in the facility were above the national average. Although considering the mean number of new treatments and the low removal of animals, the high percentage of treatments in pre-weaned calves may be related to mild cases. The severity of the disease was not recorded in the farm management software; therefore, the inclusion of mild cases in the analysis may have masked an association between late gestation maternal lipid mobilization and calf health. Additionally, a previous study of our group determined that calves exposed to maternal metabolic stress in the last 28 days of gestation had a greater basal inflammatory response and less robust response to LPS-induced inflammation during the first month of life (7). It is noteworthy to mention that metabolic stress is characterized by excessive lipid mobilization, which can lead to an abnormal inflammatory response (3). These findings indicate a potential impairment of the calf's immune response early in life. However, our results did not provide the evidence to support that calves exposed to ELM had a greater risk of developing clinical diseases in the pre-weaned period. Despite the different conditions of these 2 studies and that we did not measure the inflammatory response in pre-weaned calves. We speculate that the increase in basal inflammation in the first 4 weeks of life reported by Ling et al. (7) might not have been sufficient to result in a difference in clinical disease in the first 80 days of life, as our calves were also exposed to maternal metabolic stress.

Calves exposed to prenatal ELM had a reduced birth weight in the context of similar dry period lengths between groups. Consistent with our result, it has been reported a difference in birth weight ranging from 1.9 to 5.7 kg for calves exposed to different prenatal stressors, such as nutritional restriction or heat stress in the last week of gestation (7, 9, 27). Even though the pathway behind the difference in birth weight in our study is unknown, it has been described that 60% of fetal growth occurs in the last 60 days of gestation (28). For instance, a reduction in fetal growth may result from a disruption in the blood flow to the fetus, due to an altered metabolic status of the dam in the late gestation (11, 29, 30). This disruption might lead to a re-distribution of nutrients affecting the placental size and the diffusion of nutrients and oxygen to the fetus growth, as it has been described for maternal nutritional restriction in the late gestation (11, 29). Similarly, the metabolic status of the cow with ELM is compromised; therefore, this pathway should be considered to explain the lower birth weight of the offspring. Finally, we did not determined statistical differences for weaning weight or ADG. It is noteworthy that only 13.2% of the weaned calves had records for weaning weight, a *post-hoc* power calculation (31, 32) revealed a power of 13.9% for the association between maternal NEFA and weaning weight, leading to a minimum sample size of 64 animals per group. The reduced number of calves with weaning weight might have influenced the lack of association between maternal lipid mobilization and calf performance. Thus, studies including a greater number of animals are encouraged to establish the potential association.

### 4.2. Heifer reproductive performance

We determined an association between maternal ELM with increased age at first AI and reduced hazard for the first service. Nonetheless, this difference was only 12 days, and the age at first breeding (PLM = 400d, ELM = 412d) was within the recommendations for Holstein heifers (33). The potential mechanism for the difference in age at first service is unknown. However, the length of the difference in the age at first service was not large enough to impact the age at calving. The median age of calving (PLM = 697d; ELM = 693d) for the enrolled heifers did not differ, and it was within 23 months of life, which is in accordance with the recommendation for reproductive standards in dairy heifers (33). In addition, there is no extensive evidence to suggest an association between late gestation stressors and heifers' reproductive benchmarks. It was described that exposure to prenatal nutritional restriction in the second and third trimester of gestation did not impair the ovulation rate in the adult ewe (34) or the age at puberty of the heifer calf (35). Moreover, heifer calves exposed to heat stress in the late gestation period did not differ in the age at first service, age at calving, or in the risk of conception up to the third lactation compared to their cooled counterparts (9, 10). These late gestation stressors had a different origin than the exposure to ELM. However, similar to our results, no effect has been reported on reproductive parameters to this day.

### 4.3. First lactation health and milk yield

We did not find differences in the likelihood of treatment for diseases in the first lactation by NEFA category. It is necessary to mention that the low number of treatments in the enrolled animals difficulted the determination of a potential association. Moreover, evidence of the long-term effects of prenatal stress on disease presentation in the adult bovine is scarce. Nonetheless, the use of mice models and epidemiological studies in humans have provided valuable information to establish an association between adverse intrauterine conditions with an increase in the incidence of hypertension, abnormal glucose metabolism, excess adipose tissue deposition in the offspring's adulthood (36, 37). The conditions leading to changes in the long-term health of the offspring were described as alterations in the structure and function of vital organs and fetal genome linked to maternal undernutrition during gestation (37, 38). These findings may not be extrapolated to bovine under the conditions of our study. However, they provide evidence of the importance of the uterine environment in long-term health. Studies addressing the health of the offspring after exposure to late gestation prenatal stressors are encouraged for bovines.

We detected no differences in 305ME by maternal NEFA category. There are no studies assessing the exposure to prenatal ELM on milk production of the offspring, and limited studies have evaluated the effect of stressors in the last week of gestation on productive performance. It has been reported that adverse intrauterine conditions may induce epigenetic changes in the fetal genome that might reduce milk yield (39–41). When comparing cows exposed to *in utero* heat stress or cooling in the last 45 d of gestation, it was determined a reduction of 1.3–5.1 kg/d of milk in the first 84 and 245 d of lactation, respectively (9, 10, 41). However, it is important to note that even though ELM and heat stress are both maternal stressors, the exposure to heat stress is usually longer in duration that the one from ELM, which could explain some of the differences between both stressors. Moreover, the mechanisms associated with reduced milk production could differ greatly between stressors. However, we cannot dismiss the hypothesis that prenatal exposure to maternal ELM might be associated with production in adulthood.

### 4.4. Removal from the herd

The dairy farm enrolled in this study had 2.5% of calf removal for the pre-weaned period. Even though our estimate for animals leaving the herd in the pre-weaned stage included deaths and sells, it was lower than the 5.0% reported for pre-weaned mortality as national average (26) despite our incidence of diseases being higher than reported in these national statistics. However, the data for latest national dairy health was collected in a different time (year 2014) to our study (years 2015–2020) and included other US regions beyond Michigan, thus not making it fully representative for comparisons. Additionally, a statistical comparison between our groups was unsuitable due to the reduced number of ELM calves removed from the herd at this stage. Thus, it was not possible to elucidate a potential association between prenatal exposure to ELM and pre-weaned mortality risk in our study. Contrasting our results, the exposure to maternal undernutrition in the last third of gestation in beef calves has been associated with increased pre-weaned mortality of 4.9 and 7.0% (27, 42). However, in beef calves part of these differences might be explained by a reduction in the dam's milk production, the length of the pre-weaned period, and not exclusively for exposure to prenatal undernutrition. Therefore, these results should be interpreted with caution, as a straightforward comparison is not adequate due to the production system and type of stressor.

In addition, there is evidence suggesting that animals exposed to prenatal stressors may have a reduced probability of survival in adulthood (40, 43, 44). We assessed the likelihood of survival only until the first calving; therefore, it is unknown if there is an association between ELM and longevity beyond that reproductive benchmark. Notably, heifer calves exposed to *in-utero* heat stress had a reduced probability of survival to the first calving of 11% and 19.5% compared to calves exposed to thermoneutral prenatal conditions (9, 10). However, the mechanism behind the differences in survival may differ from prenatal exposure to ELM.

### 4.5. Limitations

To our knowledge, this is the first study attempting to determine an association between maternal ELM and the offspring's health and performance. The cut-off used for prepartum ELM is currently accepted for detrimental effects on the health and performance of the transition cow (5). However, this threshold has not been tested as an appropriate indicator of the offspring's health and performance. Moreover, this study was conducted with the records of one farm. The advantage of this approach is the uniformity in management practices, such as detection and treatment of diseases, reproductive management of the heifer, and animal removal decisions. However, the main disadvantage of using a single farm is that the results cannot be generalized to other dairy operations. Also, there was a considerable amount of missing data due to some performance parameters (e.g., weights) not being recorded for all calves. This could lead to biased estimates due to missing data patterns. However, we explored the data to find parameters of missingness associated to year, season, parity of the dam, sex, etc., not finding clear patterns in the records.

In addition, we determine a low prevalence of ELM in the last 2 weeks of gestation. Prepartum concentrations of NEFA usually show a skewed distribution (45), and this was evident in our results (Figure 2). Thus, resulting in a critical difference in the number of animals in the two categories of NEFA used in the study, which difficulted the data analysis. Furthermore, the sample size of this study was determined by the availability of records and not by sample size calculation. Moreover, we lost over 50% of animals at weaning due to the removal of bull calves from the analysis. We might, therefore, lack the power to demonstrate our hypothesis. We performed a *post-hoc* power calculation (31, 32) for the association between maternal plasma NEFA category and respiratory treatment in the pre-weaned life and 305ME in the first lactation, we determined a power of 18.1% and 25.1% for those variables, respectively. Therefore, we cannot exclude a potential association between maternal excessive lipid mobilization and the offspring's long-term health and performance. Studies assessing the cut-off with a balanced distribution of calves in the lipid mobilization groups and with multisite data are encouraged.

## 5. Conclusion

In this study, the current cut-off for excessive maternal lipid mobilization prepartum was associated with a reduced birth weight, first breeding hazard and with a longer median age at first breeding in the female offspring. However, no associations were detected between maternal NEFA groups with the hazard of pre-weaned diseases, pre-weaned ADG, the number of AI, age at first calving, odds of disease in the first lactation, 305ME in the first lactation, or culling from birth until the first calving.

## Data availability statement

The raw data supporting the conclusions of this article will be made available by the authors, without undue reservation.

## Ethics statement

Exemption from protocol review was granted by the Michigan State University Institutional Animal Care and Use Committee because only electronic records of the farm were utilized.

## Author contributions

AA, JB, KS, and ES collected the data. AA conceived and designed the study. AV-M cleaned and analyzed the data. AV-M drafted the manuscript with supervision from AA. All authors reviewed and approved the final version of the manuscript.
